# MiR-27b directly targets Rab3D to inhibit the malignant phenotype in colorectal cancer

**DOI:** 10.18632/oncotarget.23237

**Published:** 2017-12-12

**Authors:** Yang Luo, Shi-Yong Yu, Jian-Jun Chen, Jun Qin, Yi-Er Qiu, Ming Zhong, Min Chen

**Affiliations:** ^1^ Department of Gastrointestinal Surgery, Ren Ji Hospital, School of Medicine, Shanghai Jiao Tong University, Shanghai 200127, P.R. China; ^2^ Department of General Surgery, Shanghai Pudong New Area People's Hospital, Shanghai 201200, P.R. China

**Keywords:** colorectal cancer, miR-27b, Rab3D, prognosis

## Abstract

MiRNAs, as oncogenes or as anti-oncogenes, play critically regulated roles in the initiation and progression of colorectal cancer at posttranscriptional level. However, the underlying functions of miR-27b in colorectal cancer remain largely unexplored. Here, we demonstrated miR-27b is significantly down-regulated in colorectal cancer tissues, and decreased miR-27b expression was closely associated with shorter overall survival of patients with colorectal cancer. By gain- and loss-of-function studies, we showed miR-27b remarkably suppressed cell proliferation and invasion of colorectal cancer. Furthermore, luciferase reporter assay identified Rab3D was the direct functional target of miR-27b. And Rab3D partly reversed the suppression of cell proliferation and invasion caused by miR-27b mimics. Finally, the animal experiment showed miR-27b plays a crucial role on colorectal cancer progression by targeting Rab3D. Taken together, our study implied miR-27b inhibits cell growth and invasion by targeting Rab3D, and miR-27b is a potential biomarker for prognosis and therapeutic target in colorectal cancer.

## INTRODUCTION

Colorectal cancer (CRC) is one of the most common gastrointestinal malignancy and the five leading causes of cancer-related death in China [1, 2]. Despite significant advancements in diagnosis and treatment for this disease, especially surgery, chemotherapy and radiotherapy, the prognosis of CRC patients is still extremely poor because of recurrence and distant metastasis [3, 4]. Thus, there is an urgent need for the identification of carcinogenesis and development factors and understanding the molecular mechanisms underlying CRC.

MicroRNAs (miRNAs) are a class of noncoding RNAs that can trigger either translational repression or mRNA degradation by targeting the 3′ untranslated region (3′-UTR) of specific mRNAs [5, 6]. They have been demonstrated to play an important role in the development and progression of various cancers [7]. MiRNA-27b is an important member of miRNA family. Previous study has shown miR-27b could inhibit promoter methylation and target HMGB3 mRNA to modulate tamoxifen resistance and epithelial-mesenchymal transition (EMT) in breast cancer cells [8]. Goto et al. found miR-27b plays a vital role of anti-tumor to inhibit cell proliferation, migration and invasion, and may serve as a reliable prognostic biomarker in in prostate cancer [9]. Tao et al. found miR-27b exerts tumor-suppressive effects in gastric cancer through the suppression of oncogene ROR1 expression [10]. However, the role that miR-27b plays in colorectal cancer remains largely unknown.

In this study, we examined miR-27b expression in a large cohort of colorectal cancer tissue specimens and found miR-27b is tightly associated with overall survival of CRC patients. Furthermore, *in vitro* experiments showed that miR-27b inhibited growth and invasion of colorectal cancer cells through targeting Rab3D. Finally, *in vivo* experiments showed miR-27b plays a crucial role on CRC progression by targeting Rab3D. Thus, these data provide new insight into molecular mechanisms underlying progression of colorectal cancer.

## RESULTS

### MiR-27b expression is significantly down-regulated in CRC

**Figure 1:**
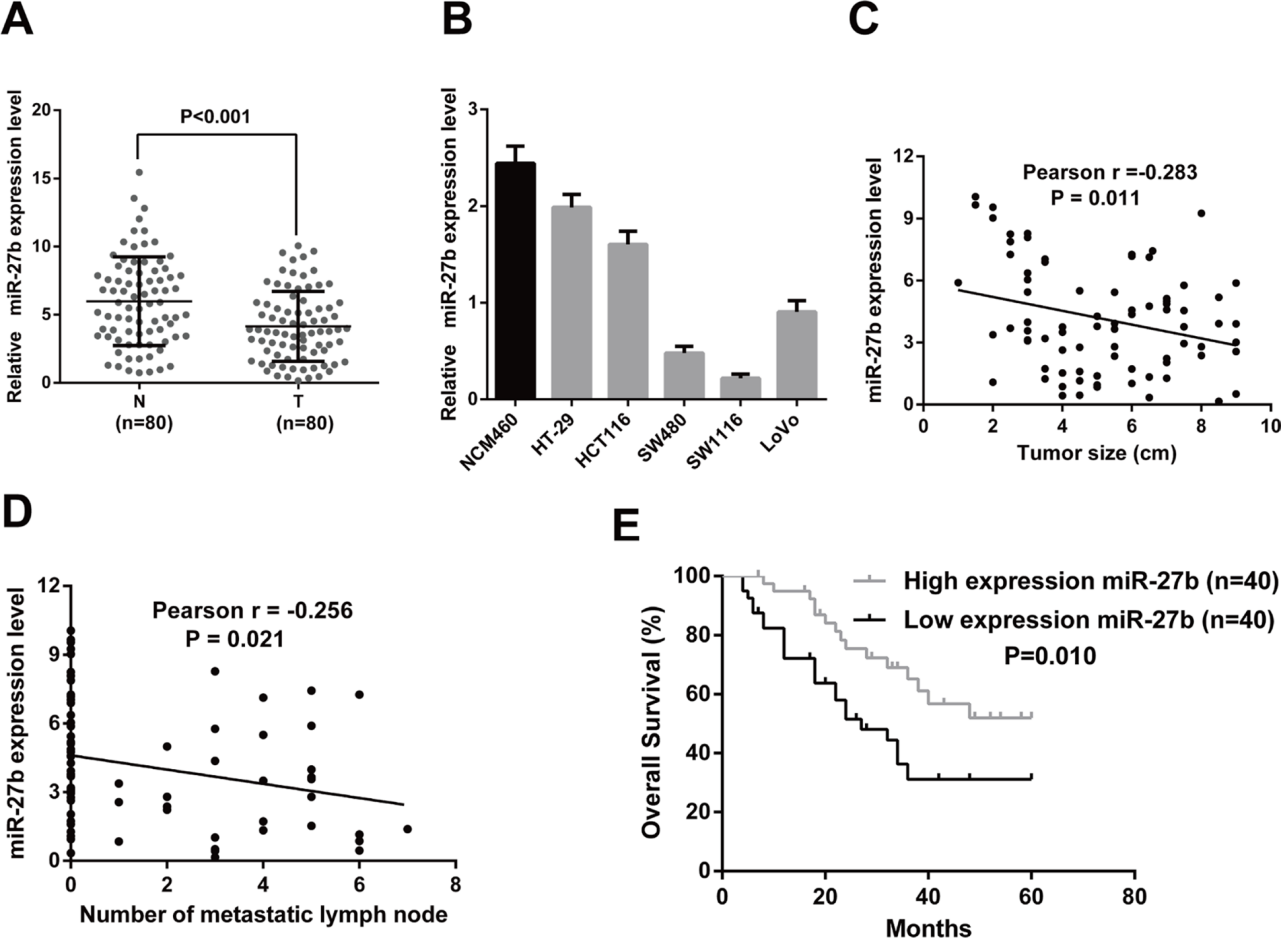
MiR-27b is down-regulated in CRC and correlated with prognosis of CRC patients (**A**) The transcription level of miR-27b in 80 matched CRC tissues (T) and adjacent normal tissues (N) by RT-qPCR. (**B**) Histograms of the transcription level of miR-27b in CRC cell lines and the normal colonic epithelial cells NCM460. (C–D). The relationship between miR-27b and clinicopathologic characteristics, like tumor size (**C**), number of metastatic lymph nodes (**D**). (**E**) Comparisons of the overall survival duration between the low and high miR-27b expression. Results shown are the mean ± SEM of triplicate determination from three independent experiments.

To elucidate the expression pattern of miR-27b in CRC, we first detected the expression level of miR-27b in 80 matched CRC tumor and non-tumor tissues by RT-qPCR. The results showed the expression level of miR-27b was obviously down-regulated in CRC tissues compared with their corresponding normal counterparts (Figure 1A). Then, we measured the expression level of miR-27b in five CRC cell lines and the normal colonic epithelial cell line, NCM460, and found the highest miR-27b level was detected in the NCM460 cells (Figure 1B). Furthermore, we tried to explore the relationship between clinical features and miR-27b expression levels, in our case, a negative correlation was found between tumor size and miR-27b expression levels (r = −0.283, *P* = 0.011, Figure 1C), as well as number of metastatic lymph nodes and miR-27b expression levels (r = −0.256, *P* = 0.021, Figure 1D). These findings imply a potential role in tumor growth and metastasis of miR-27b in colorectal cancer.

### Elevated miR-27b expression predicts a poor prognosis in patients with CRC

To determine the clinical significance of miR-27b, we analyzed the association between miR-27b expression and the clinicopathological characteristics of CRC. As shown in Table 1, the level of miR-27b was negatively associated with tumor size (*P* = 0.041) and tumor node metastasis (TNM) stage (*P* = 0.004). However, no significant associations were found between miR-27b expression and other clinical features, including age, gender, CEA, and tumor location.

**Table 1:** Correlations between miR-27b expression and clinicopathologic features in 80 colorectal cancer patients

Clinicopathological feature		Expression of miR-27b		
	Total 80	Low (*n =* 40, 42.67%)	High (*n =* 40, 57.33%)	*P* value (χ^2^ test)
**Age (years)**				
< 65	42	20 (47.62)	22 (52.38)	0.654
≥ 65	38	20 (52.63)	18 (47.37)	
**Gender**				
Male	39	21 (53.85)	18 (46.15)	0.502
Female	41	19 (46.34)	22 (53.66)	
**Tumor location**				
Rectum	41	20 (40.98)	21 (59.02)	0.461
Colon	39	20 (45.30)	19 (54.70)	
**CEA level**				
≤ 5 ng/ml	41	22 (53.66)	19 (46.34)	0.502
> 5 ng/ml	39	18 (46.15)	21 (53.85)	
**Tumor size**				
≤ 5 cm	47	28 (59.57)	19 (40.43)	**0.041**
> 5 cm	33	12 (36.36)	21 (63.64)	
**TNM stage(AJCC)**				
Stage I–II	41	14 (34.15)	27 (65.85)	**0.004**
Stage IV–III	49	26 (66.67)	13 (33.33)	

Values in parentheses indicate percentage values. The bold number represents the *P*-values with significant differences.

To determine the prognostic value of miR-27b for CRC, the relationship between miR-27b expression and the clinical follow-up data were analyzed using Kaplan-Meier survival curves and the log-rank test. The results revealed high expression of miR-27b was positively associated with overall survival (OS) (*n* = 80, *P* = 0.001, Figure 1E), which indicates that OS is better in CRC patients with high miR-27b expression than in those with low miR-27b expression.

To directly identify the risk factors associated with OS in CRC patients, univariate and multivariate analyses were performed to confirm that miR-27b represents an independent risk factor for poor prognosis. Univariate Cox regression analysis showed that the miR-27b expression level, tumor size, age and TNM stage were significantly associated with OS (Table 2). Furthermore, multivariate Cox regression analysis confirmed the miR-27b expression level and TNM stage were independent predictors of OS in patients with CRC (Table 2). These data indicated high expression of miR-27b may be a predictor for the diagnosis and prognosis of colorectal cancer patients.

**Table 2:** Univariate and multivariate analyses of prognostic parameters for survival in 80 colorectal cancer patients

	Univariate analysis			Multivariate analysis		
Prognostic parameter	HR	95% CI	*P* value	HR	95% CI	*P* value
**Expression of miR-27b** (low vs. high)	0.437	0.226–0.845	**0.014**	0.448	0.224–0.896	**0.023**
**Age** ( < 65 vs. ≥ 65)	1.075	0.568–2.033	**0.049**	0.823	0.426–1.590	0.562
**Gender** (male vs. female)	1.131	0.596–2.147	0.707	-	-	-
**Tumor Size** ( ≤ 5 cm vs. > 5 cm)	2.384	1.216–4.677	**0.012**	1.999	1.001–3.992	0.050
**CEA level** (≤ 5 ng/ml vs. > 5 ng/ml)	1.207	0.624–2.333	0.576	-	-	-
**Tumor location** (rectum vs. colon)	1.189	0.624–2.266	0.599	-	-	-
**TNM stage** (I vs. II vs. III vs. IV)	1.827	1.300–2.566	**0.001**	1.683	1.181–2.399	**0.004**

HR: Hazard ratio; CI: Confidence interval. The bold number represents the *P*-values with significant differences.

### miR-27b inhibits cell proliferation and invasion in CRC cells *in vitro*

**Figure 2:**
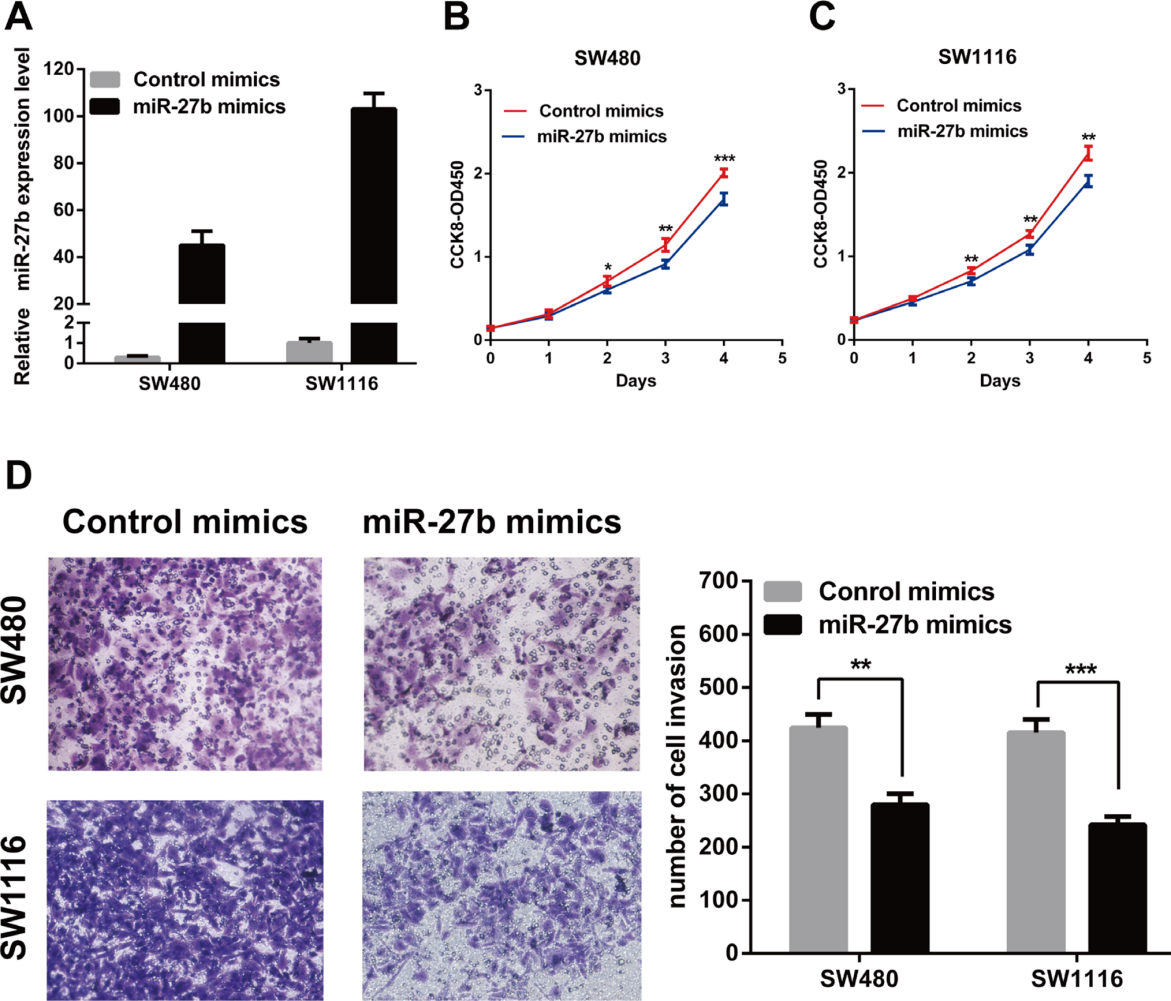
MiR-27b inhibits cell proliferation and invasion in CRC cells (**A**) miR-27b overexpression efficiency was confirmed by RT-qPCR in CRC cells. (**B**–**C**) miR-127b mimics inhibited proliferation in SW480 and SW1116 cells detected by CCK-8 assays. (**D**) miR-127b mimics significantly decreased the invasive potential of CRC cells by Transwell assays. Results shown are the mean ± SEM (^*^P < 0.05, ^**^P < 0.01, ^***^P < 0.001) of triplicate determination from three independent experiments.

**Figure 3:**
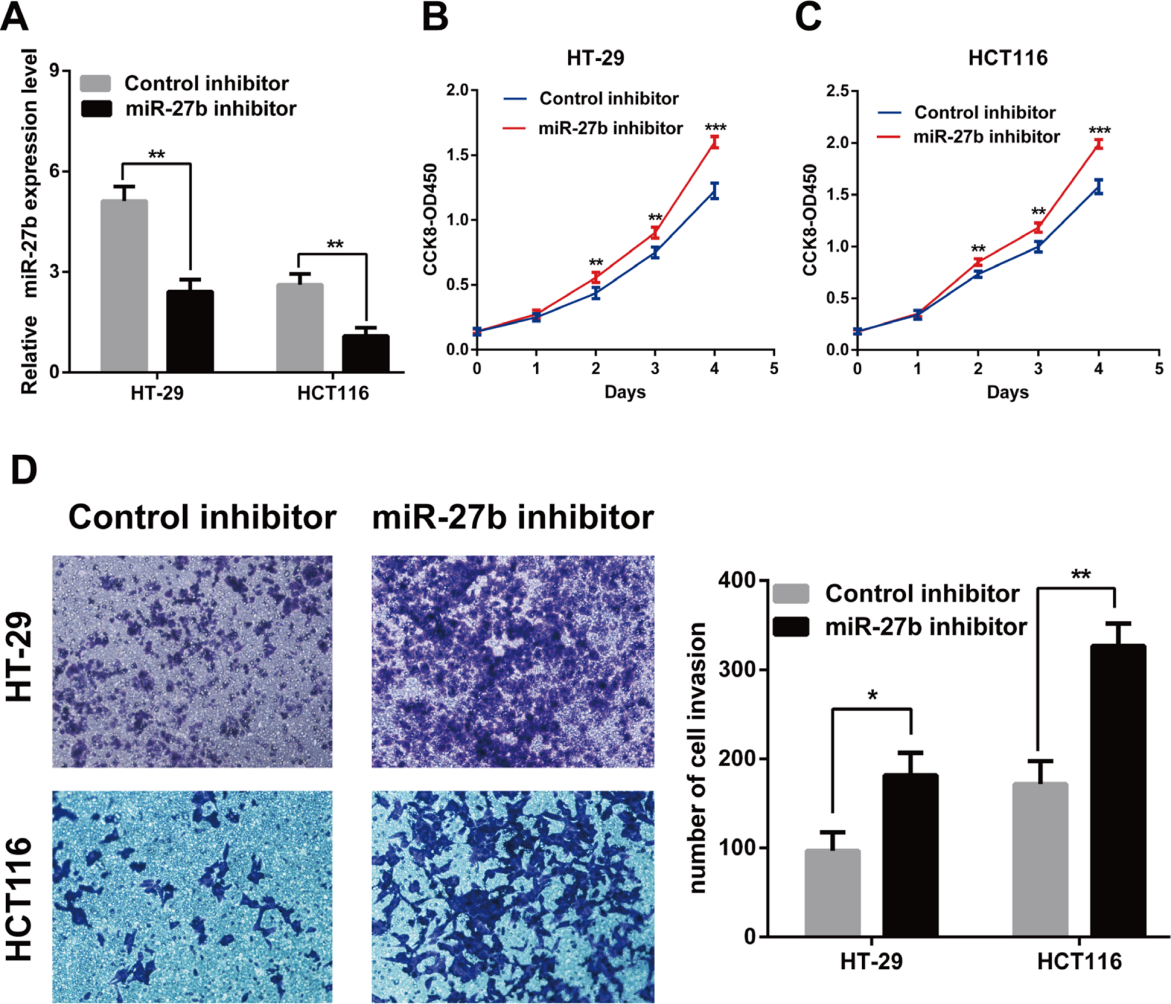
Knockdown of miR-27b promotes cell proliferation and invasion in CRC cells (**A**) miR-27b knockdown efficiency was confirmed by RT-qPCR in CRC cells. (**B**–**C**) miR-27b inhibitor promoted proliferation in HT-29 and HCT116 cells detected by CCK-8 assays. (**D**) miR-27b inhibitor significantly increased the invasive potential by Transwell assay. Results shown are the mean ± SEM (^*^P < 0.05, ^**^P < 0.01, ^***^P < 0.001) of triplicate determination from three independent experiments.

To explore the role of endogenous miR-27b in colorectal cancer, miR-27b was over-expressed in the SW480 and SW1116 cells or inhibited in the HT-29 and HCT116 cells by transiently transfecting with miR-27b mimics or miR-27b imhibitors. The transfection efficiency was confirmed by RT-qPCR (Figure 2A and Figure 3A).

To test whether miR-27b inhibited CRC cell proliferation and invasion, we performed CCK-8 assay and transwell assay in CRC cells. The results indicated that that the proliferation and invasion of SW480 and SW1116 cells with miR-27b mimics were significantly lower than those of the control (Figure 2B–2D), while the proliferation and invasion of HT-29 and HCT116 cells with miR-27b inhibitor were significantly enhanced (Figure 3B–3D). Our data indicated that miR-27b can inhibit cell proliferation and invasion in colorectal cancer cells *in vitro*.

### Rab3D is a direct target of miR-27b

**Figure 4:**
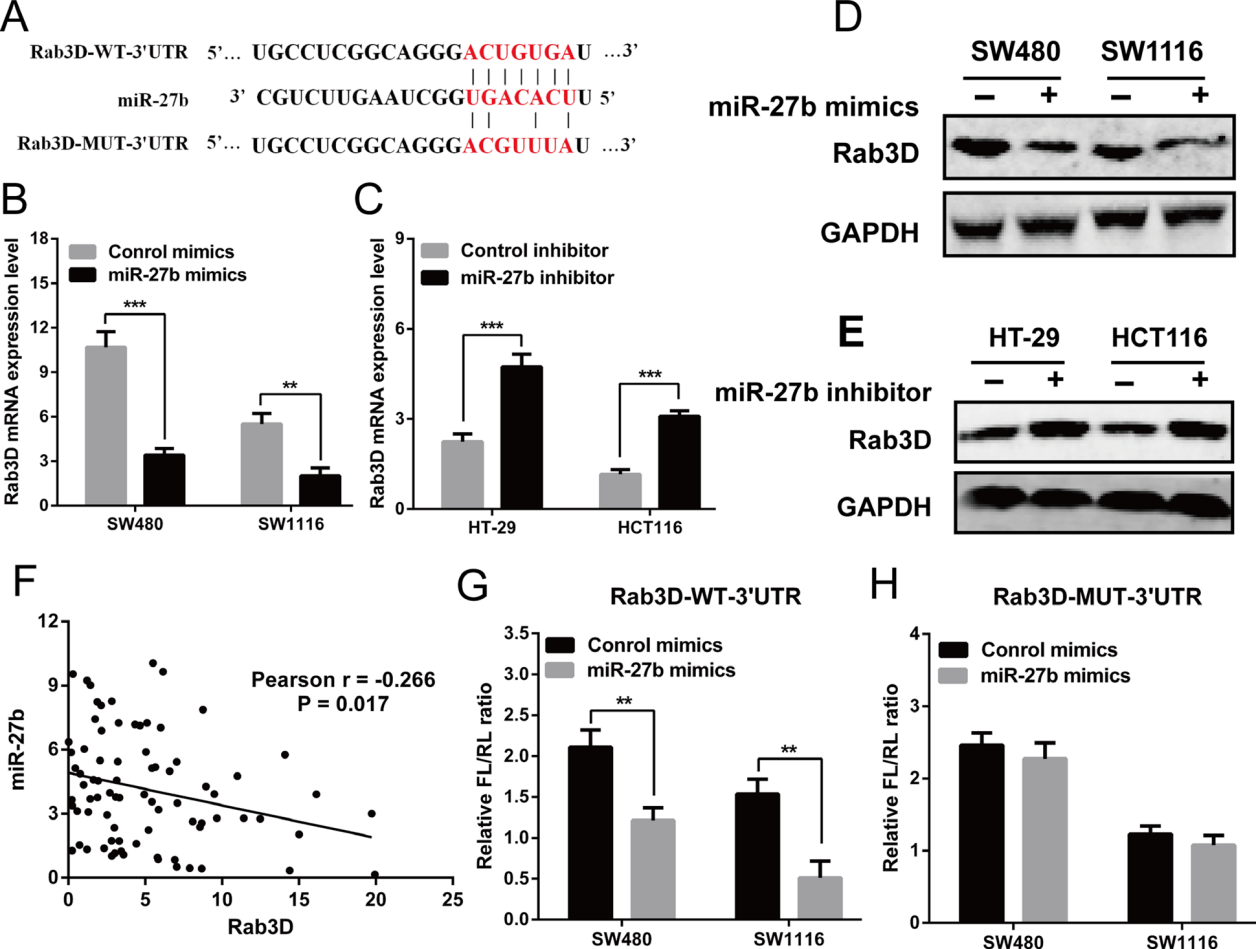
Rab3D is a direct target of miR-27b (**A**) The predicted sites of miR-27b binding to the 3′-UTR region of Rab3D were detected using bioinformatics prediction tools. The mutated site in the 3′-UTR region of Rab3D was also shown (**B**–**E**). The mRNA and protein level of Rab3D were detected in the presence of miR-27b mimics (**B**–**D**) or miR-193b inhibitor (**C**, **E**) in CRC cells. (**F**) Spearman’s correlation analysis between miR-27b and Rab3D mRNA levels in 80 CRC patients (**G**–**H**). The luciferase activity after transfection with the luciferasereporter plasmid containing either wild type or mutant Rab3D 3′-UTR in the presence of miR-27b mimic or negative controls in SW480 and SW1116 cells. Results shown are the mean ± SEM (^**^P < 0.01, ^***^ *P* < 0.001) of triplicate determination from three independent experiments.

To investigate the mechanism of miR-27b in colorectal cancer, we screened the target genes of miR-27b by using TargetScan (http://www.targetscan.org/), miRDB (http://www.mirdb.org/miRDB/) and picTar (http://pictar.mdc-berlin.de/), and found that Rab3D was identified as a candidate (Figure 4A). Then, RT-qPCR and Western blot analysis found over-expression of miR-27b inhibited Rab3D expression, and knockdown of miR-27b increased Rab3D expression (Figure 4B–4E). Interestingly, miR-27b levels were found to be markedly inversely correlated with Rab3D expression (*r* = −0.266, *P* = 0.017, Figure 4F).

To verify whether miR-27b can bind to the predicted site of Rab3D, a human Rab3D 3′ untranslated region (3′UTR) fragment containing the wild-type or mutant miR-27b-binding site was inserted downstream of the luciferase open reading frame (Figure 4A). The luciferase reporter assay showed the relative luciferase activity was markedly decreased after co-transfection with pmirGLO/Rab3D-WT-3′UTR and miR-27b in SW480 and SW1116 cells, compared with control (Figure 4G). Whereas the luciferase activity was unaffected after co-transfection with pmirGLO/Rab3D-MUT-3′UTR and miR-27b (Figure 4H). The results indicated that miR-27b can specifically binds to the 3′UTR of Rab3D mRNA.

### Rab3D expression is significantly up-regulated in CRC

**Figure 5:**
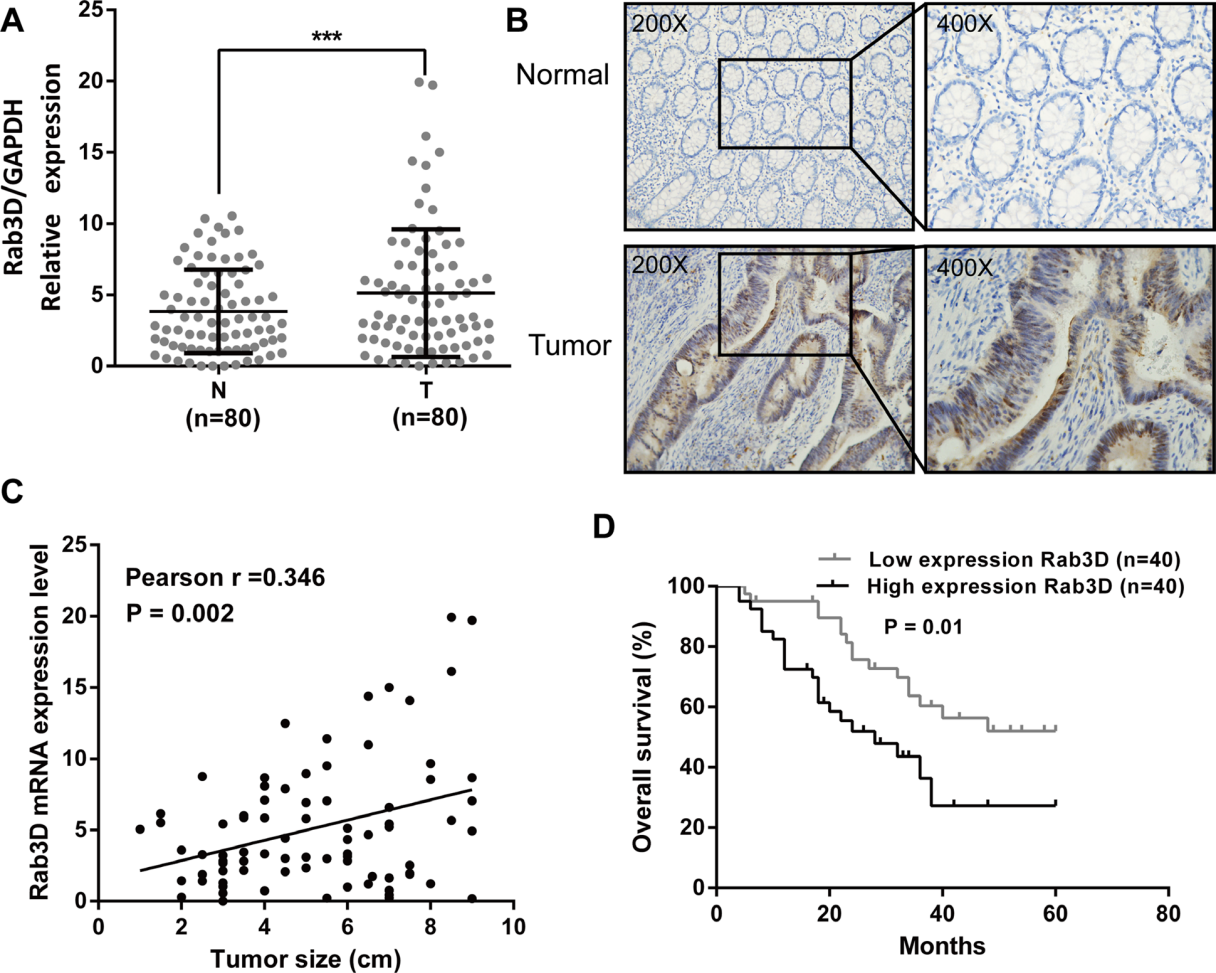
Rab3D is up-regulated in CRC and develop the prognosis of CRC patients (**A**) The transcription level of Rab3D in 80 matched CRC tissues (T) and adjacent normal tissues (N) by RT-qPCR. (**B**) Rab3D expression in CRC was determined by immunohistochemistry. (**C**) The relationship between Rab3D and tumor size in CRC patients. (**D**) Comparisons of the overall survival duration between the low and high Rab3D expression. Results shown are the mean ± SEM of triplicate determination from three independent experiments.

To evaluate the expression status of Rab3D in CRC tissues, we examined the expression level of Rab3D in 80 matched CRC tumor and non-tumor tissues by RT-qPCR and immunohistochemical staining. The result showed that the expression level of Rab3D was significantly increased in colorectal cancer compared with corresponding normal counterparts (Figure 5A–5B). Furthermore, a positive correlation was found between Rab3D and tumor size expression levels (r = 0.346, *P* = 0.002, Figure 5C). Finally, we found overall survival is poorer in CRC patients with high Rab3D expression than in those with low Rab3D expression (*P* = 0.01, Figure 5D).

### Restoration of Rab3D abolishes the tumor suppressor role of miR-27b

**Figure 6:**
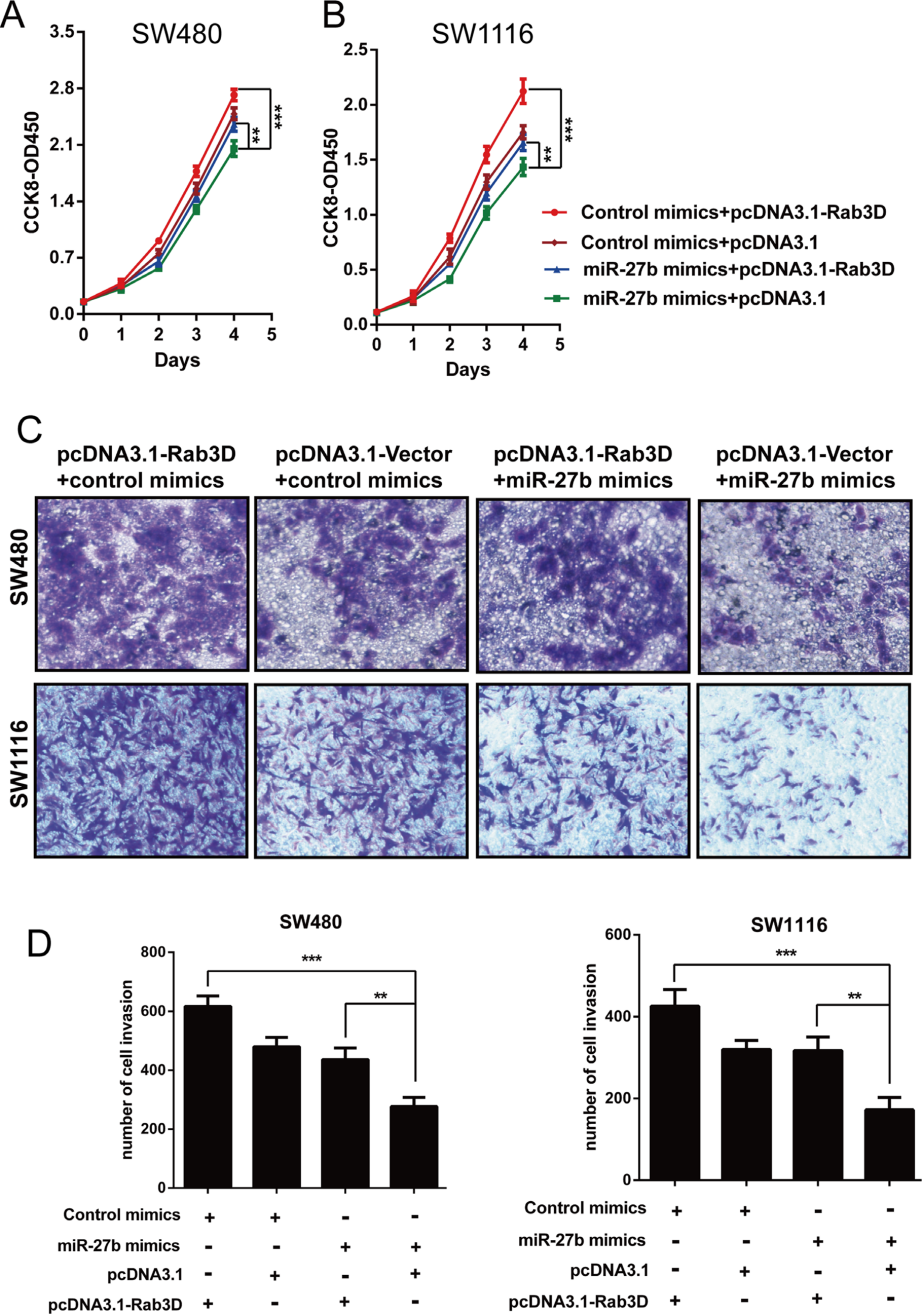
miR-27b contributes to cell proliferation and invasion by inhibiting Rab3D The implication of miR-27b mimics on the proliferative or invasive potential of CRC cells was detected in the presence of pcDNA3.1-Rab3D treatment by CCK-8 assays (**A**–**B**) or trasnwell assays (**C**–**D**). Results shown are the mean ± SEM (^**^P < 0.01, ^***^ *P* < 0.001) of triplicate determination from three independent experiments

To further illustrate that miR-27b affects cell proliferation and invasion by inhibiting Rab3D expression, we investigated whether Rab3D counteracted the suppression of cell phenotypes caused by miR-27b overexpression in CRC cells. SW480 and SW1116 cells were cotransfected with miR-27b mimics and either Rab3D or pcDNA3.1 empty vector. The data clearly confirmed that ectopic expression of Rab3D partly reversed the suppression of cell proliferation (Figure 6A–6B) and invasion (Figure 6C–6D) caused by miR-27b overexpression in CRC cells. These data collectively indicated that miR-27b inhibits cell proliferation and invasion by targeting Rab3D, and that miR-27b may act as a “tumor suppressor” in colorectal cancer.

### MiR-27b by targeting Rab3D inhibited tumor growth *in vivo*

**Figure 7:**
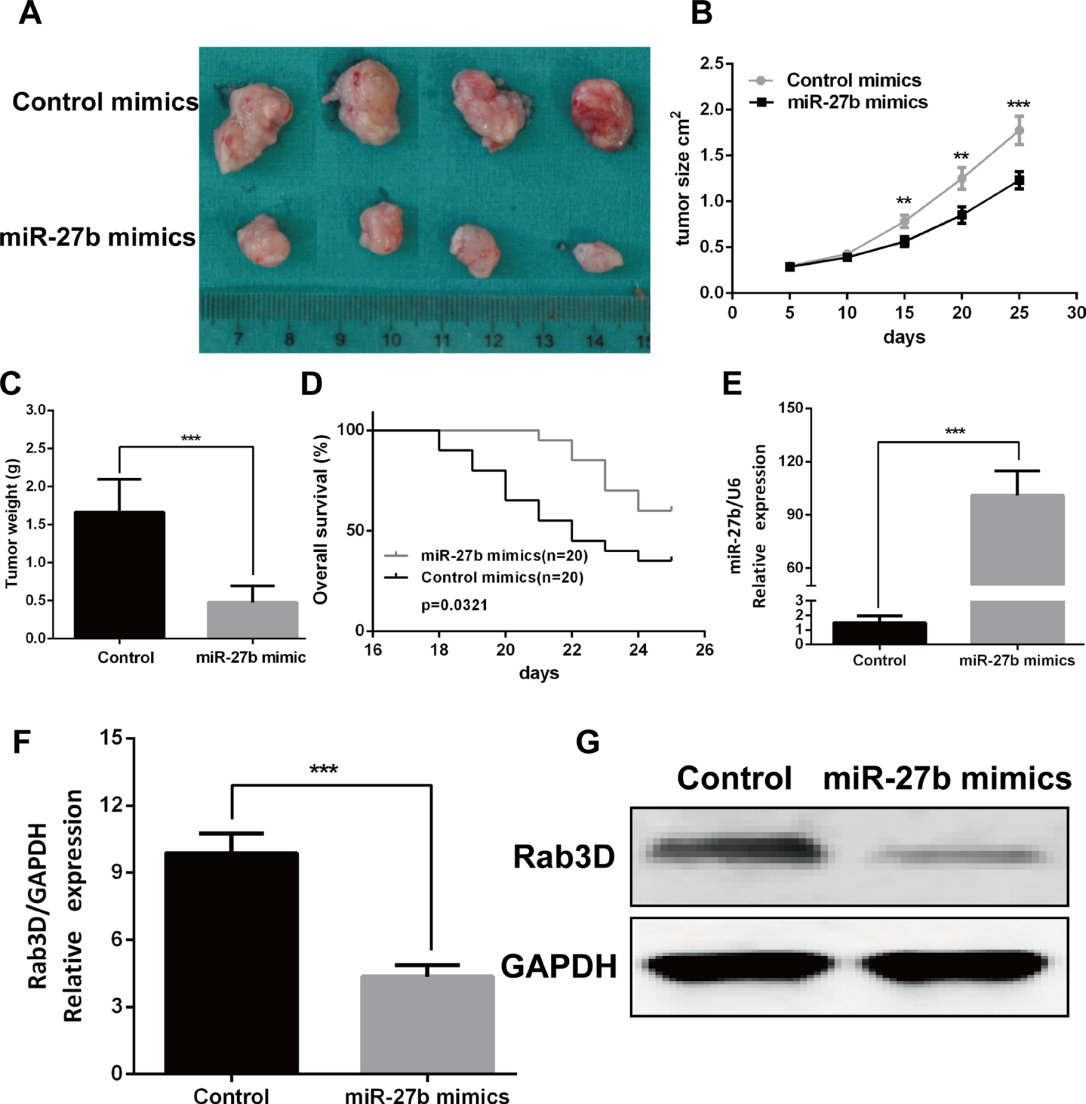
miR-27b inhibited tumor growth *in vivo* Tumor xenograft volume (**A**) and weight (**C**) in miR-27b–treated nude mice was smaller than that in the control. (**B**) miR-27b–treated nude mice was better overall survival than the control group. (**D**) miR-27b expression in miR-27b–treated nude mice and Control group. (**E**–**F)**. Rab3D expression in the miR-127b–treated nude mice were decreased compared with that in control group (**G**). Results shown are the mean ± SEM (^**^P < 0.01, ^***^P < 0.001).

To verify the effects of miR-27b on tumorigenesis *in vivo*, miR-27b agomir or control agomir was directly injected into the CRC implanted tumor, and found miR-27b significantly decreased tumor growth (Figure 7A–7C), not reduced tumor metastasis (date not showed). We also found overall survival is better in mice with high miR-27b expression than in those with low miR-27b expression (*P* = 0.0321, Figure 7D). And the expression of miR-27b was significantly increased in the miR-27b treated with mice (Figure 7E). Furthermore, the mRNA and protein expression of Rab3D was markedly decreased in the miR-27b treated with mice compared with that in control (Figure 7F–7G). Taken together, these results demonstrate that miR-27b plays a crucial role on CRC progression by targeting Rab3D.

## DISCUSSION

Accumulating evidence indicates that Dysregulation of miRNAs is connected with initiation and progression of CRC, since they may serve as oncogenes or tumor suppressors [11]. For example, Up-regulation of miR-183 significantly inhibited cell autophagy and apoptosis through targeting of UVRAG in colorectal cancer [12]. miR-331-3p can decrease HER2 expression to suppress proliferation and promote apoptosis through the PI3K/Akt and ERK1/2 pathways in colorectal cancer [13]. Similarly, miR-27b has been found in a poor prognostic phenotype of human gastric cancer, which is due to the regulatory effect on invasive growth and metastasis [10, 14]. Therefore, it is reasonable to hypothesize miR-27b may play a role in the pathogenesis of CRC patients. In the current study, We found miR-27b was down-regulated in CRC tissues, and negatively correlated with tumor size and TNM stage (*P* < 0.05), and patients with a low level of miR-27b expression had significantly shorter survival times. Importantly, Univariate and multivariate analysis suggested miR-27b expression was an independent risk factor for poor outcome of CRC patients.

Previously, increased expression of down-regulation of miR-27b predicts the better prognosis has been reported in clear cell renal cell carcinoma (ccRCC), which demonstrated down-regulated miR-27b is associated with higher tumor grade and stage, and plays a tumor-suppressive role [15]. However, up-regulation of miR-27b in breast cancer correlated with a poor prognostic group, and promote cell cell proliferation and tumor progression [16, 17]. These findings suggested the expression pattern functions of miR-27b may be tissue specific. Consistent with the observation in ccRCC, we also demonstrated miR-27b acts as a tumor suppressor in CRC in cell proliferation and invasive capacity. Our results showed that the exogenous overexpression of miR-27b inhibited proliferative and invasive ability of CRC cells. These findings imply miR-27b may be function as tumor suppressor in CRC.

Rab3D, belonging to a member of the Ras superfamily of monomeric G proteins, localize to distinct cellular compartments and promote specific steps of intracellular membrane trafficking [18, 19]. Our previous studies found Rab3D expression correlated with more advanced pathological grade and associated with reduced overall survival, which suggest Rab3D might be involved in CRC progression and metastasis [20]. Furthermore, it has been reported that Rab families participated in the process of miRNA-related regulation [21–23]. Therefore, we selected Rab3D for further study. As expected, we confirmed that Rab3D mRNA and protein were down-regulated by the ectopic expression of miR-27b. Then, luciferase reporter assay identified that miR-27b could directly bind to the 3′ UTR of Rab3D. Moreover, the animal experiment showed miR-27b plays a crucial role on CRC progression by targeting Rab3D.

The oncogenic activities of Rab3D have been reported in many human cancers, including colorectal cancer [20], breast cancer [24] and pancreatic cancer [25]. Despite several studies emphasized Rab3D can promote tumor progression by targeting with signaling pathways, the precise molecular mechanism for the functional role of Rab3D in cancer remains elusive. In colorectal and breast cancer, Rab3D has been reported to promote metastasis through activating Akt/GSK3β/Snail pathway and inducing epithelial mesenchymal transition (EMT) process [20, 26]. However, whether Akt/GSK3β/Snail signaling pathways is involved in the anti-oncogenic activities of miR-27b in CRC remain further investigation.

In summary, our study revealed miR-27b inhibited the growth and invasion of colorectal cancer cells by targeting Rab3D, and lower expression of miR-27b was associated with poor prognosis and was an independent predictor of patients’ survival. These findings uncover a novel molecular mechanism underlying colorectal cancer growth and metastasis. More importantly, miR-27b might be a novel biomarker for diagnosis and prognosis and a potential therapeutic target in colorectal cancer.

## MATERIALS AND METHODS

### Patients and samples

Eighty pairs of human colorectal cancer and adjacent non-cancerous colorectal tissues (not less than 5 cm away from the tumor) were collected from Renji Hospital (Shanghai, China) from January 2011 to December 2015. The follow-up duration was calculated from the date of surgery to the date of death or the last known follow-up. None of these cases had received radiotherapy, chemotherapy, hormone therapy or any other related anti-tumor therapy before surgery. All tumor samples were independently reviewed by two pathologists to confirm the diagnosis of adenocarcinoma. The median follow up of patients was 29.04 months (ranging from 4 to 60 months). And according to the median value, the CRC tissues were divided into two groups: relative high miR-27b expression group (*n* = 40) and relative low miR-27b expression group (*n* = 40). All samples were obtained following the participants’ written informed consent, and all experiments were approved by the local ethics committee of the Shanghai Jiao-Tong University School of Medicine at Renji Hospital.

### Cell culture and transfection

Human colorectal cancer cell lines, HT-29, HCT116, SW480, SW1116 and LoVo were purchased from the Institute of Biochemistry and Cell Biology at the Chinese Academy of Sciences (Shanghai, China). The normal colonic epithelial cell line NCM460 was purchased from American Type Culture Collection (ATCC). All cells were grown in Dulbecco’s modified Eagle’s medium (DMEM) (Gibco, USA) supplemented with 10% fetal bovine serum (Gibco, USA) and 100 U/ml penicillin and 100 mg/ml streptomycin in a humidified atmosphere of 5% CO_2_ at 37°C.

miR-27b mimics, miR-27b inhibitors and respective negative control oligonucleotides were obtained from GenePharma (Shanghai, China). Transfection was performed with Lipofectamine 2000 reagent (Invitrogen, USA) following the manufacturer’s protocol.

### Total RNA extraction and real-time quantitative PCR

Total RNA was extracted from CRC tissues and cells using Trizol reagent (Takara, Japan). The cDNA was synthesized using a microRNA Reverse Transcription Kit (Promega, USA) or a PrimeScript RT-PCR kit (Takara, Japan), respectively. RT-qPCR was performed using StepOne Real-Time PCR System (Applied Biosystems, USA). Primers of miR-27b and U6 were obtained from GeneCopoeia (California, USA). The primers for Rab3D were 5′-TGGTGGGGAACAAGTGTGAC-3′ and 5′-GGAATGAGCCATGCAGGAGT-3′, and for GAPDH were 5′-TGAAGGTCGGAGTCAACGGA-3′ and 5′-CCTGGAAGATGGTGATGGGAT-3′. The expression of Rab3D was normalized with GAPDH. Melting curve analysis was carried out for each PCR reaction to confirm the specificity of amplification, and the fold change was calculated using the 2^−ΔΔCT^ method.

### Cell proliferation assay

A density of 3,000-3,500 indicated CRC cells/well upon different treatments, transfected with miR-27b mimics or miR-27b inhibitors, were seeded in a 96-well cell culture plate, grown at 37°C overnight. After transfection at 1d, 2d, 3d, 4d, the medium was replaced with 100 ul of fresh medium and 10 ul CCK-8 (Dojindo, Japan) working solution was added to each well. Then cells continued to be incubated at 37°C for 1 h, and the absorbance was detected at 450 nm with a microplate reader (Model 680, BioRad, USA). Each experiment was performed in quadruplicate determination from three independent experiments.

### Cell invasion assay

The invasive ability of CRC cells was detected by transwell model (Corning, USA) according to the manufacturer’s instructions. Briefly, 5 × 10^4^ cells suspended in serum-free medium were plated on the top of each chamber, while medium containing 20% FBS was put in the lower chamber. After incubating for 48 h, the chambers were disassembled, the non-invaded cells that remained on the upper chamber were removed, and the membranes were stained with a 2% crystal violet solution for 30 min and placed on a glass slide. Then, cells that had migrated across the membrane were counted in five random visual fields using a light microscope. Each experiment was performed in triplicate determination from three independent experiments.

### Western blotting

All proteins were resolved on a 10% SDS-PAGE and were then transferred onto a PVDF membrane. Membranes were incubated with blocking buffer for 90 min at room temperature and then incubated with an antibody against Rab3D (1:1000, Abcam, UK) or GAPDH (1:1000, Abcam, UK) overnight at 4°C. The membranes were washed and incubated with a horseradish peroxidase (HRP)-conjugated secondary antibody. Protein expression was assessed by enhanced chemiluminescence and exposure to chemiluminescent film. The LabWorks image acquisition and analysis software was used to quantify band intensities.

### Luciferase reporter assay

The wild-type Rab3D 3′UTR, containing putative binding sites for miR-27b, was inserted into pmir-GLO dual-luciferase vector (Genearray Biotechnology, Shanghai, China), and mutant Rab3D 3′UTR was generated based on the pMIR- Rab3D -3′UTR by mutating 3 nt. The reporter plasmid was transiently transfected into SW480 and SW1116 cells in the presence of either miR-27b mimics or control mimics. After 48 h, the cells were harvested and lysed, and luciferase activity was measured using the Dual-Luciferase Reporter Assay System (Promega, USA) according to the manufacturer’s instructions. Each experiment was performed in quadruplicate determination from three independent experiments.

### Animal experiment

Xenograft tumors were generated by subcutaneous injection of 4×10^6^ cells on the hind limbs of BALB/C athymic nude mice (nu/nu) obtained from the Animal Center of East China Normal University, Shanghai, China. All mice were housed and maintained under specific pathogen-free conditions. When the average value of tumor sizes was up to 100 mm^3^, the mice were separated into 2 groups randomly, one with subcutaneous injection of miR-27b (Agomir) at different sites, and the other with Control (Agomir). Injection was performed every five day. All mice were euthanized at 25 days after the initial injection, and the tumors were excised.

### Statistical analyses

Data were expressed as the means ± SEM of at least three independent experiments. All statistical analyses were performed using the SPSS 19.0 software. Overall survival rate was calculated according to the Kaplan-Meier method and the difference in survival curves was evaluated by the log-rank test. The Student’s *t*-test was used to analyze differences between two groups. *P* values less than 0.05 were considered statistically significant.
